# Association Between Subjective Symptoms, Psychological Stress and Quality of Life Among Older Japanese Adults

**DOI:** 10.3390/geriatrics11050123

**Published:** 2026-09-04

**Authors:** Sachie Ono, Akira Komatsuzaki, Yukika Enoki, Takeshi Kamoda, Chikage Kato, Kiyoka Arashi

**Affiliations:** 1 Department of Preventive and Community Dentistry, The Nippon Dental University, Niigata 951-8580, Japan; sachie@ngt.ndu.ac.jp (S.O.); kamoda-t@ngt.ndu.ac.jp (T.K.); 2 Department of Dental Hygiene, The Nippon Dental University College at Niigata, Niigata 951-8580, Japan; enoki11@ngt.ndu.ac.jp (Y.E.); kiyoka@ngt.ndu.ac.jp (K.A.); 3 Department of Dental Technology, The Nippon Dental University College at Niigata, Niigata 951-8580, Japan; chikage@ngt.ndu.ac.jp

**Keywords:** older adults, national statistics, subjective symptoms, psychological stress

## Abstract

**Background/Objectives**: A crucial issue in Japan, as a rapidly aging society, is supporting the quality of life (QOL) of older adults to ensure they do not require long-term care. This study aims to examine the association between subjective symptoms and psychological stress and QOL decline using a large-scale Japanese national statistics database. **Methods**: Anonymized data were analyzed from 23,649 adults aged ≥ 65 years, obtained from the Comprehensive Survey of Living Conditions carried out in 2019 in Japan. The degree of association of different factors with QOL decline was analyzed using univariate symptom analysis and adjusted odds ratios (ORs) obtained through binary logistic regression. We also compared the reported frequency and mean number of symptoms in a group with poor QOL and one with good/regular QOL. **Results**: In the group with poor QOL, significantly more individuals were aged ≥ 75 years, had high levels of psychological stress (Kessler Psychological Distress Scale [K6]), and reported subjective symptoms (*p* < 0.01). The mean number of symptoms was significantly higher in this group (5.77) than in the good/regular QOL group (3.34, *p* < 0.01). In the binary logistic regression, the variables with the highest adjusted ORs were the K6 group (4.49), impaired limb movement (2.81), and numbness of the limbs (2.01). **Conclusions:** These findings indicate that different symptoms, including stress, have varying impacts on QOL in older adults. Addressing symptoms associated with reduced QOL in community-dwelling older adults may help promote continued aging in place.

## 1. Introduction

Japan has become a super-aged society, shifting the focus of its healthcare initiatives from living longer to living better. Efforts are now underway to implement measures that improve the quality of life (QOL) of older adults to prevent dependence on care and establish comprehensive community-based care systems [1]. The World Health Organization defines QOL as an individual’s perception of their position in life in relation to the culture and value systems under which they live and their goals, expectations, standards, and concerns [2]. Therefore, QOL is considered multifactorial, comprising subjective factors such as perceived well-being and health. While subjective symptoms are based on individual perception, they can have a direct negative impact on QOL and, as such, are a major factor in poorer perceived health [3]. As subjective health is sensitive to declines in QOL, it was employed in this study as a QOL scale [4].

The onset of subjective symptoms is thought to be one of the body’s physiological response processes, although previous reports have suggested that there are individual differences in the likelihood of perceiving them [5] and that some diseases may tend to go unnoticed because their symptoms are not readily perceived. Among dental diseases, for example, periodontal disease is widely referred to as a “silent disease” [6]. The mechanisms of onset of a wide variety of subjective symptoms remain unclear, and only a few fields, such as palliative care, have prioritized research that focuses on their effects on QOL and everyday life [7].

The perception of subjective symptoms is likely to be strongly influenced by psychological stress, as we highlighted in previous studies [8,9]. The effect of a given symptom on QOL may vary depending on whether it is acute, chronic, mild, or severe. These factors may also determine whether patients seek institutional medical care and their motivation to practice self-care, thus impacting the prognosis of the disease. In some cases, subjective symptoms may contribute more to disease diagnosis than objective clinical examination [10], and they are thus emphasized in the clinical practice guidelines used in many settings [11,12,13]. Many clinicians infer diagnoses based on a wide range of information, including both subjective symptoms and objective findings, and medical interviews can be used to match subjective symptoms with a diagnosis [14].

Recently, increasing interest has been shown in multimorbidity, where multiple chronic diseases are present together and may cause a variety of symptoms [15]. A systematic review covering both developed and developing countries reported that the prevalence of multimorbidity increased with age [16]. In China, the prevalence of multimorbidity among older adults (age ≥ 80 years) is reported to be >50% [17]. In Japan, studies of disease co-occurrence patterns have started to emerge, including our previous study [18].

However, comparing multimorbidity between countries is difficult because there are large disparities in the definition of multimorbidity between studies, as well as in the subjects and diseases studied [19]. In Japan, no consensus has been reached on a definition of multimorbidity, and it is not a recognized diagnostic term. As a result, polypharmacy researchers recently introduced the UK National Institute for Health and Care Excellence guidelines [20] to improve the efficiency of medical care for the elderly.

In this context, we previously conducted descriptive epidemiological studies that explored multimorbidity in Japan by using national statistics databases to examine the impact of subjective symptoms from different perspectives [21,22,23]. The results of those studies suggested that the degree of impact of subjective symptoms on QOL, everyday life, and psychological stress varies. The participants, however, were mainly people of working age, so the situation among older adults remains unclear.

Given this background, in the present study, we merged two anonymized national statistics databases to obtain the largest dataset currently available and used these data to conduct a comprehensive analysis of the impact of subjective symptoms and stress on QOL in older adults.

## 2. Materials and Methods

### 2.1. Study Design and Participant Data

This cross-sectional study made use of the Statistics Act, through which the Ministry of Health, Labour and Welfare (MHLW) in Japan can make anonymized national statistical data available for scientific research. They provide tabulated data that have been processed to ensure they are not personally identifiable. With the cooperation of the MHLW, we merged the anonymized A and B data files from the Comprehensive Survey of Living Conditions to create the largest dataset on older adults currently available.

**Figure 1:**
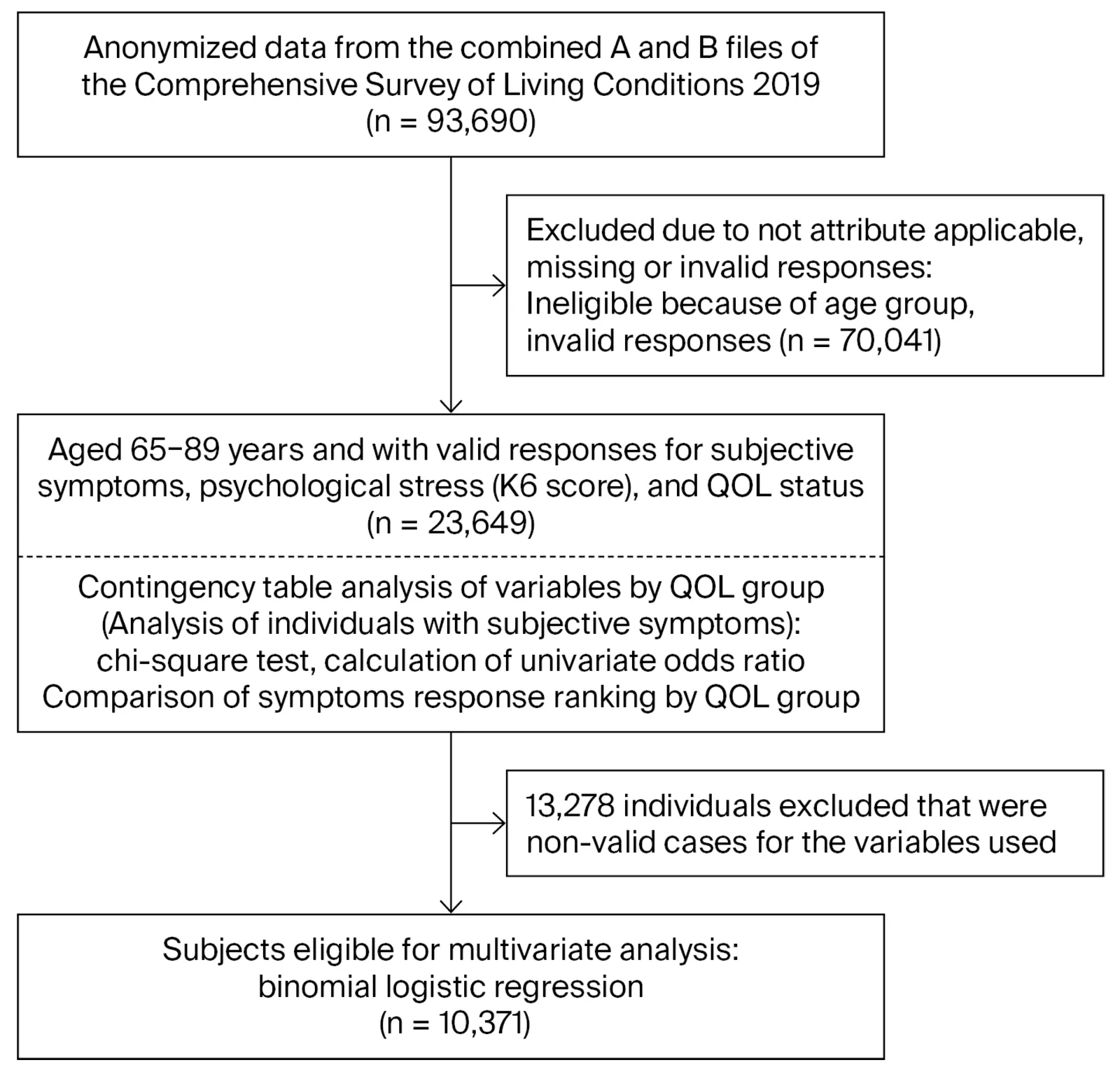
Data flow for the analysis of anonymized data.

The Comprehensive Survey of Living Conditions asks households about their income, health, and daily lives to help the government improve social welfare policies. Detailed, large-scale surveys are conducted at three-year intervals. The combined dataset comprises a total of 93,690 individuals, from which data for 23,649 older adults aged 65–89 years were used for analysis in the present study. The data analysis procedures used in this study are outlined in Figure 1.

In the analysis, a five-point rating of QOL (answer to the question “What is your current state of health?”) was used to group subjects into a poor QOL group and a good/regular QOL group using the criteria of Yokoi et al. [22] (Table 1), and the two groups were compared. Subjects were also classified for analysis based on their Kessler Psychological Distress Scale (K6) scores into a high stress group (K6 score ≥ 10) and a low stress group (K6 score < 10), according to the criteria of Komatsuzaki et al. [23].

### 2.2. Contingency Table Analysis (Univariate Analysis) Comparing Gender, Other Variables, and Subjective Symptoms by QOL Group

Survey items were compared between the QOL groups using a contingency table analysis (univariate analysis by means of 2 × 2 contingency tables). Chi-square tests were used to examine the associations between the QOL group and various basic attributes, such as gender, the K6 psychological distress group, and the most frequently reported subjective symptoms (the top 12 out of 39 symptoms). Then, the univariate odds ratios (ORs) and 95% confidence intervals were calculated.

### 2.3. Comparison of Subjective Symptom Rankings by QOL Group

The Wilcoxon signed-rank test was used to compare the response frequency for all 39 subjective symptoms between the two QOL groups. The data used for the comparison comprised the number of responses for each symptom in each QOL group.

In addition, the mean number of reported symptoms was calculated for each QOL group and then compared between groups using Welch’s *t*-test.

### 2.4. Multivariate Analysis with the QOL Group as the Dependent Variable (Binary Logistic Regression)

To examine the variables that were found to be significantly associated with the QOL in the contingency table analyses, a binary logistic regression analysis (forced entry method) was performed, with the QOL group as the dependent variable and gender, age, household structure, K6 score group, and presence of subjective symptoms as adjustment variables. Then, the adjusted ORs were calculated.

### 2.5. Statistical Analysis

Data were aggregated using Microsoft Office 2019 Excel (Microsoft Japan, Tokyo, Japan). Chi-square tests, univariate OR calculations, the Wilcoxon signed-rank test, and binary logistic regression were performed using BellCurve for Excel Ver.4.10 (BellCurve, Tokyo, Japan). The level of significance was set at *p* < 0.05 for all statistical tests.

### 2.6. Ethical Considerations

This study was approved by the Ethical Review Board of the Nippon Dental University College at Niigata (approval date: 12 June 2025, ID No. NDUC-127) and is published with the permission of the MHLW (Government Statistics 0616 No.2), based on Article 36 of the Statistics Act. This study was carried out in accordance with the Declaration of Helsinki, and all procedures for the protection of personal information complied with the Ethical Guidelines for Epidemiological Research issued by the Ministry of Education, Culture, Sports, Science and Technology and the MHLW. The authors obtained anonymized tabular data files from the MHLW and used these data for the analyses. The dataset comprised only responses from participants who had provided informed consent.

## 3. Results

### 3.1. Results of Contingency Table Analysis Comparing Gender, Age, and Other Variables by QOL Group

Table 2 shows the results of the contingency table analysis comparing gender, age, household structure, K6 score group, and the presence of subjective symptoms by QOL group. Significant differences in age, K6 score group, and the presence of subjective symptoms were found between groups (*p* < 0.01): the poor QOL group contained a higher proportion of adults aged ≥ 75 years (26.1%), those with a K6 score ≥ 10 (high stress group, 58.9%), and those with subjective symptoms (38.3%).

High univariate ORs were observed for the high stress group (7.54) and the presence of subjective symptoms (11.31), indicating that stress and subjective symptoms have a major impact on QOL.

### 3.2. Results of the Comparison of Subjective Symptom Rankings by QOL Group

The contingency table analysis compared the most frequently reported subjective symptoms between QOL groups, from the top-ranked symptom (lower back pain, 40.3%) to the 12th-ranked symptom (cough/phlegm, 15.3%). The results are shown in Table 3.

For all 12 subjective symptoms, the proportion of the poor QOL group that reported experiencing each symptom was higher than the proportion of those who did not, and the result of the chi-square test showed this difference to be significant (*p* < 0.01). Five subjective symptoms were reported by >50% of the poor QOL group; namely, impaired limb movement (66.5%), numbness of the limbs (58.4%), cough/phlegm (51.9%), forgetfulness (51.8%), and difficulty seeing objects (51.4%).

In addition, the univariate ORs were significant for all 12 symptoms. The symptoms with an OR > 2 were impaired limb movement (3.98) and numbness of the limbs (2.71).

### 3.3. Comparison of Subjective Symptom Rankings by QOL Group

The 10 most frequently reported subjective symptoms in each QOL group are reported in Table 4. Differences between the two QOL groups for each of the 39 subjective symptoms were examined using the Wilcoxon signed-rank test.

In the poor QOL group, symptoms reported most frequently included impaired limb movement (ranked 3rd) and numbness of the limbs (ranked 4th). In the good/regular QOL group, symptoms reported most frequently included blurred vision (ranked 4th).

### 3.4. Comparison of the Mean Number of Symptoms by QOL Group

The mean number of symptoms reported in both groups is shown in Table 5. The poor QOL group reported a mean value of 5.77 symptoms, and the good/regular QOL group reported a significantly lower mean value of 3.34 symptoms (*p* < 0.01). Therefore, on average, the poor QOL group reported over two more subjective symptoms than the good/regular QOL group.

### 3.5. Binary Logistic Regression with the QOL Group as the Dependent Variable

Table 6 shows the results of the binary logistic regression with the QOL group as the dependent variable. The symptoms with the highest adjusted ORs were impaired limb movement (2.70), numbness of the limbs (2.01), lower back pain (1.32), cough or phlegm (1.27), and difficulty seeing objects (1.21). These associations were all significant (*p* < 0.01).

The variable with the highest adjusted OR was the K6 group (4.55), which was inserted as an adjustment variable. This finding indicates that psychological stress has a substantial impact on QOL. The coefficient of determination, which indicates the accuracy of the analysis, was 0.14. While this is low, the model was considered appropriate.

In addition, the univariate OR for dental symptoms (1.60, Table 3) was significant (*p* < 0.01), but the adjusted OR was not (1.07).

## 4. Discussion

The results of the study allow us to clarify the effects of subjective symptoms on QOL in older adults based on a large-scale anonymized database of Japanese national statistics.

Even at the international level, few studies have simultaneously compared multiple symptoms to analyze their associations with QOL, and most of those have included a lower number of subjects and fewer symptoms. This may be because clinical researchers increasingly tend to report on subjective symptoms within their specific fields, as medical practice has become more specialized. Pati et al. [24] identified that living with multiple diseases complicates the health and well-being (happiness, health, and welfare) of older adult patients. Healthcare policy advocates and providers have raised concerns from the perspective of healthcare provision about these “complicated” patients requiring intervention by numerous different medical specialists.

In particular, bearing in mind that older adults in Japan receive care through a multidisciplinary approach that encompasses not only healthcare but also long-term care and welfare services, multimorbidity needs to receive the same level of attention as measures to prevent frailty.

The finding that we consider most noteworthy from the present study is that persons in the poor QOL group reported, on average, two more subjective symptoms than those in the good/regular QOL group. With the aging of the population and the increasing prevalence of chronic diseases, this increase in subjective symptoms is becoming a public health concern at a global level [25]. The coexistence of multiple chronic diseases is likely to complicate health screening and management. It may be assumed that, if this situation continues, there will be a deterioration in public health provision, an increase in healthcare expenditure, and widening health disparity.

However, health screening systems and clinical practice guidelines remain largely focused on addressing single diseases. Shapiro et al. [26] pointed out the need to remove the barriers between specialties in many clinical settings.

It is also noteworthy that, apart from lower back pain, subjective symptoms with a high incidence had a low association with QOL decline, whereas subjective symptoms associated with the physical function of the limbs made a greater contribution. A prior study of working-age adults [22] showed that fatigue-related symptoms such as lower back pain, stiff shoulders, and blurred vision had a greater effect on QOL, whereas in older adults, symptoms directly linked to activities of daily living had more impact.

The Japanese Ministry of Health, Labour and Welfare (MHLW) provides benefits and implements projects to prevent the need for long-term care as part of the long-term care insurance system, with an emphasis on functional rehabilitation [27]. Rather than focusing solely on conventional end-of-life palliative care, the MHLW has also taken a population-based approach to improving QOL among older adults.

It is important to note that the results of the binary logistic regression showed an adjusted OR of 4.49 for the K6 group (stress status), which was higher than that of any of the subjective symptoms. These results are consistent with the findings of previous research, indicating that the stress assessment using the K6 scale was conducted appropriately [28]. This points to a need to examine background stress factors in the living environments of older adults. Additionally, cough/phlegm showed a high adjusted OR of 1.28, and it is possible that some older adults reported coughing due to aspiration-related choking. A more detailed investigation of this finding is needed.

This study has some limitations. First, because we used data from the Comprehensive Survey of Living Conditions, we were only able to grasp a cross-sectional image of subjective symptoms “in the last few days”. This means that the findings may not provide an accurate understanding of acute symptoms, which tend to resolve within a short time. Using the results of a cross-sectional, multi-item questionnaire survey thus allowed only a limited grasp of the effect of subjective symptoms as factors reducing QOL and, as such, longitudinal studies are needed to clarify the causal relationships between subjective symptoms and QOL.

Furthermore, it is necessary to consider the potential influence of physical and social confounding factors that could not be addressed in the analysis. It is also important to note that this study uses data from before the COVID-19 pandemic, so there may be differences in the perception of symptoms and health status between the pre- and post-pandemic periods.

On the other hand, the fact that this study used a large-scale database of Japanese national statistics for analysis is a strength, because this enabled us to secure a large number of subjects compared to similar studies [29]. Because social variables are included in the household questionnaire, it will be possible to construct a variety of analytical models in future research, thereby allowing additional analyses to be conducted from different perspectives.

## 5. Conclusions

The association between subjective symptoms and reduced QOL in older adults was examined through an analysis of anonymized data from the Comprehensive Survey of Living Conditions carried out in Japan in 2019. The degree to which different factors contributed to QOL decline was analyzed by means of univariate and multivariate analyses (binary logistic regression) and by comparing the frequency and mean number of symptoms by QOL group. The results indicated that, in the poor QOL group, significantly higher proportions of individuals aged ≥ 75 years old had high levels of stress (K6) and reported subjective symptoms (*p* < 0.01). The mean number of symptoms was significantly higher in the poor QOL group (5.77) than in the good/regular QOL group (3.34, *p* < 0.01). In the binary logistic regression, the variables with the highest adjusted ORs were the K6 group (4.49), impaired limb movement (2.81), and numbness of the limbs (2.01).

These results indicate that different symptoms, including factors such as stress, have varying impacts on QOL decline in old age. Addressing symptoms associated with reduced QOL in community-dwelling older adults may help promote continued aging in place.

## Figures and Tables

**Table 1:** QOL group classification based on the perception of health.

Responses to Question	n	%	QOL Classification (*)
Poor	619	2.6	Poor QOL group (1) (n = 4508)
Quite poor	3889	16.4	
Regular	12,428	52.6	Good/regular QOL group (0) (n = 19,141)
Quite good	3890	16.4	
Good	2823	11.9	
Total	23,649	100	

* Set as a variable for binary logistic regression analysis.

**Table 2:** Contingency table comparing gender, age, and other survey items by QOL group.

Survey Item (*)	Poor QOL Group	Good/Regular QOL Group	Total	Chi-Square Test	Univariate Odds Ratio (95% CI)
Gender					0.96 (0.90–1.03)
Male (1)	2051 (18.8)	8880 (81.2)	10,931 (100.0)		
Female (0)	2457 (19.3)	10,261 (80.7)	12,718 (100.0)		
Age: years					1.94 (1.81–2.07)
Upper age group: 75–89 (1)	2530 (24.9)	7617 (75.1)	10,147 (100.0)	**	
Lower age group: 65–74 (0)	1978 (14.6)	11,524 (85.4)	13,502 (100.0)		
Household structure					0.96 (0.90–1.02)
Single-person household (1)	2695 (18.8)	11,635 (81.2)	14,330 (100.0)		
Three-generation households, parent–child households (0)	1813 (19.5)	7506 (80.5)	9319 (100.0)		
K6 group (psychological state score)					7.58 (6.83–8.41)
High stress: ≥10 (1)	986 (59.1)	682 (40.9)	1668 (100.0)	**	
Low stress: <10 (0)	3522 (16.0)	18,459 (84.0)	21,981 (100.0)		
Presence of subjective symptoms					11.55 (10.58–12.62)
Yes	3860 (37.2)	6511 (62.8)	10,371 (100.0)	**	
No	648 (4.9)	19,141 (94.8)	13,278 (100.0)		

* Set as variables for binary logistic regression analysis (n (%); **: *p* < 0.01).

**Table 3:** Comparison of subjective symptoms by QOL group (top 12 highest-ranked subjective symptoms shown).

Survey Item (*)	Poor QOL Group	Good/Regular QOL Group	Total	Chi-Square Test	Univariate Odds Ratio (95% CI)
Lower back pain					1.58 (1.46–1.71)
Yes (1)	1812 (43.7)	2338 (56.3)	4150 (100.0)	**	
No (0)	2048 (32.9)	4173 (67.1)	6221(100.0)		
Limb joint pain					1.50 (1.37–1.64)
Yes (1)	1157 (44.4)	1449 (55.6)	2606 (100.0)	**	
No (0)	2703 (34.8)	5062 (65.2)	7765 (100.0)		
Stiff shoulders					1.25 (1.14–1.37)
Yes (1)	1044 (41.2)	1487 (58.8)	2531 (100.0)	**	
No (0)	2816 (35.9)	5024 (64.1)	7840 (100.0)		
Blurred vision					1.48 (1.34–1.64)
Yes (1)	874 (44.9)	1074 (55.1)	2174 (100.0)	**	
No (0)	2986 (35.5)	5437 (64.5)	8423 (100.0)		
Frequent urination					1.56 (1.41–1.73)
Yes (1)	891 (45.9)	1049 (54.1)	1940 (100.0)	**	
No (0)	2969 (35.2)	5462 (64.8)	8431 (100.0)		
Difficulty hearing					1.68 (1.52–1.86)
Yes (1)	906 (47.4)	1004 (52.6)	1910 (100.0)	**	
No (0)	2954 (34.9)	5507 (65.1)	8461 (100.0)		
Dental symptoms					1.57 (1.42–1.74)
Yes (1)	875 (46.1)	1023 (53.9)	1898 (100.0)	**	
No (0)	2985 (35.2)	5488 (64.8)	8473 (100.0)		
Numbness of the limbs					2.71 (2.44–3.01)
Yes (1)	981(57.4)	728 (42.6)	1709 (100.0)	**	
No (0)	2879 (33.2)	5783(66.8)	8662 (100.0)		
Forgetfulness					1.96 (1.76–2.17)
Yes (1)	859 (50.8)	831 (49.2)	1690 (100.0)	**	
No (0)	3001 (34.6)	5680 (65.4)	8681 (100.0)		
Difficulty seeing objects					1.85 (1.67–2.06)
Yes (1)	815 (49.8)	822 (50.2)	1637 (100.0)	**	
No (0)	3045 (34.9)	5689 (65.1)	8734 (100.0)		
Impaired limb movement					3.98 (3.56–4.45)
Yes (1)	1050 (65.3)	559 (34.7)	1609 (100.0)	**	
No (0)	2810 (32.1)	5952 (67.9)	8762 (100.0)		
Cough/phlegm					1.64 (1.47–1.83)
Yes (1)	744(47.4)	826 (52.6)	1570 (100.0)	**	
No (0)	3116 (35.4)	5685 (64.6)	8801 (100.0)		

* Set as variables for the binary logistic regression analysis (n (%); **: *p* < 0.01).

**Table 4:** Comparison of subjective symptom response rankings by QOL group (top 10 most frequent symptoms shown).

Response Rank	Poor QOL Group	n (%)	Good/Regular QOL Group	n (%)	Total	n (%)
1st	Lower back pain	1812 (46.9)	Lower back pain	2338 (35.9)	Lower back pain	4150 (40.0)
2nd	Limb joint pain	1157 (30.0)	Stiff shoulders	1487 (22.8)	Limb joint pain	2606 (25.1)
3rd	Impaired limb movement	1050 (27.2)	Limb joint pain	1449 (22.3)	Stiff shoulders	2531 (24.4)
4th	Stiff shoulders	1044 (27.0)	Blurred vision	1074 (16.5)	Blurred vision	1948 (18.8)
5th	Numbness of the limbs	981 (25.4)	Frequent urination	1049 (16.1)	Frequent urination	1940 (18.7)
6th	Difficulty hearing	906 (23.5)	Dental symptoms	1023 (15.7)	Difficulty hearing	1.910 (18.4)
7th	Frequent urination	891 (23.1)	Difficulty hearing	1004 (15.4)	Dental symptoms	1898 (18.3)
8th	Dental symptoms	875 (22.7)	Tinnitus	849 (13.0)	Numbness of the limbs	1709 (16.5)
9th	Blurred vision	874 (22.6)	Forgetfulness	831 (12.8)	Forgetfulness	1690 (16.3)
10th	Forgetfulness	815 (21.1)	Cough/phlegm	826 (12.7)	Difficulty seeing objects	1637 (15.8)
Wilcoxon signed-rank test *		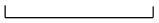 **				

* Performed with all 39 symptoms, ranked, for both QOL groups. The number of subjects in each group was used to calculate n (%). (**: *p* < 0.01).

**Table 5:** Comparison of the mean number of symptoms between groups.

QOL Group	Mean ± SD (Number of Symptoms: Range)	Welch’s *t*-Test
Poor QOL group	5.77 ± 4.52 (1–33)	**
Good/regular QOL group	3.34 ± 2.69 (1–24)	
Total	4.24 ± 3.68 (1–33)	

SD: Standard deviation. The number of subjects in each group was used to calculate the mean (**: *p* < 0.01).

**Table 6:** Results of binary logistic regression with the QOL group as the dependent variable.

Explanatory Variable	Variable	Adjusted Odds Ratio	95% CI
	K6 group	4.49 **	3.92–5.15
	Impaired limb movement	2.81 **	2.48–3.17
	Numbness of the limbs	2.01 **	1.79–2.26
	Lower back pain	1.33 **	1.21–1.45
	Cough/phlegm	1.28 **	1.13–1.44
	Age	1.22 **	1.12–1.34
	Difficulty seeing objects	1.22 **	1.07–1.38
	Frequent urination	1.17 **	1.04–1.31
	Gender	1.14 **	1.05–1.25
	Difficulty hearing	1.14 *	1.02–1.29
	Forgetfulness	1.14 *	1.01–1.29
	Limb joint pain	1.11 *	1.00–1.23
Classification accuracy	discrimination rate	70.3%	
Coefficient of determination	R^2^ (Cox–Snell)	0.14	

Gender and age were included as adjustment variables. Only significant adjusted odds ratios are shown. (**: *p* < 0.01,*: *p* < 0.05).

## Data Availability

The original contributions presented in the study are included in the article; further inquiries can be directed to the corresponding author.

## Abbreviations

The following abbreviations are used in this manuscript:

QOL	Quality of life
K6	Kessler Psychological Distress Scale—6 items
ORs	Odds ratio
MHLW	Ministry of Health, Labour and Welfare
95%C.I.	95% confidence interval
SD	Standard deviation
